# The effect of dietary lipid quality in early life on serum LysoPC(18:2) levels and their association with adult blood glucose levels in intrauterine growth restricted rats

**DOI:** 10.1186/s12986-021-00614-8

**Published:** 2021-11-27

**Authors:** Andrea Kodde, Mona Mischke, Maryam Rakhshandehroo, Jenny Voggel, Gregor Fink, Eva Nüsken, Manfred Rauh, Eline M. van der Beek, Jörg Dötsch, Kai-Dietrich Nüsken

**Affiliations:** 1 Danone Nutricia Research, Utrecht, The Netherlands; 2 Department of Pediatrics and Adolescent Medicine, Medical Faculty, University Hospital Cologne, University of Cologne, Cologne, Germany; 3 Department of Pediatrics and Adolescent Medicine, University Hospital Erlangen, Erlangen, Germany; 4 Department of Pediatrics, University Medical Centre Groningen, University of Groningen, Groningen, The Netherlands

**Keywords:** Metabolomics, Lysophosphatidylcholine, Intrauterine growth restriction, Postnatal diet, Blood glucose, Diabetes susceptibility, Milk fat globule membrane

## Abstract

Being born small-for-gestational-age, especially with subsequent catch-up growth, is associated with impaired metabolic health in later-life. We previously showed that a postnatal diet with an adapted lipid droplet structure can ameliorate some of the adverse metabolic consequences in intrauterine growth-restricted (IUGR) rats. The aim of the present work was to explore possible underlying mechanism(s) and potential biomarkers. To this end, serum metabolomics was performed in postnatal day (PN) 42 and PN96 samples of the above-mentioned rat offspring, born after uterine vasculature ligation. Blood samples were collected at PN42, directly after a postnatal dietary intervention with either complex lipid matrix (CLM) or control (CTRL) diet, and at PN96 after a subsequent western-style diet (WSD). Offspring of Non-operated (NOP) dams fed CTRL in early life were included as control group. In the PN42 metabolomics data, 11 co-abundance modules of metabolites were identified, of which four were significantly correlated to adult blood glucose levels at PN96. Further analyses showed that Lysophosphatidylcholine(18:2) (LysoPC(18:2)) levels were reduced by ligation (*p* < 0.01) and restored in CLM fed animals (*p* < 0.05). LysoPC(18:2) levels at PN42 correlated inversely with adult blood glucose levels. These data indicate that early-life LysoPC(18:2) blood levels may predict adult blood glucose levels and are affected by a postnatal diet with an adapted lipid droplet structure in IUGR offspring.

**Supplementary Information:**

The online version contains supplementary material available at 10.1186/s12986-021-00614-8.

## Introduction

The early-life environment is essentially linked to adult metabolic health and obesity risk, and potential underlying mechanisms are manifold [1]. Low birth weight, specifically when followed by subsequent catch-up growth, is associated with increased risk for later-life obesity and impaired glucose tolerance [2–4]. Intrauterine growth-restriction (IUGR) is a major cause of low birth weight [5]. In line with this, rat offspring born after IUGR have a lower birthweight, accelerated postnatal growth, increased adiposity, and impaired glucose tolerance in adulthood [6]. Nutritional interventions starting in early-life, before the onset of an adverse phenotype, may reduce the risk for later-life obesity and impaired glucose tolerance. Indeed, exposure in postnatal life to a diet with a so-called complex lipid matrix (CLM, Nuturis®), i.e. comprising large, (milk)phospholipid coated lipid droplets, has been shown to ameliorate some of the adverse metabolic consequences of IUGR, such as relative visceral adiposity, high blood glucose and triglyceride levels [7]. The CLM is inspired by the architecture of human milk lipid droplets, which are larger compared to those in regular infant milk formula (IMF) and enveloped by a milk fat globule membrane (MFGM) [8].

Part of the beneficial effect of the CLM may be explained by differences in tissue fat handling and postprandial kinetics, as indicated by earlier experiments in healthy mice and men [9, 10]. The aim of the present work was to further explore potential mechanisms and identify biomarkers underlying the beneficial effects of postnatal exposure to CLM on adult visceral adiposity, blood glucose and triglyceride levels in IUGR rats [7]. We hypothesized that differences in adult adiposity, blood glucose and triglyceride levels might arise from or could be predicted by differences in metabolites directly after exposure to the CLM. Therefore, serum metabolites were analyzed, and comprehensive data analysis was performed on collected data.

## Methods

### Study design

**Fig. 1:**
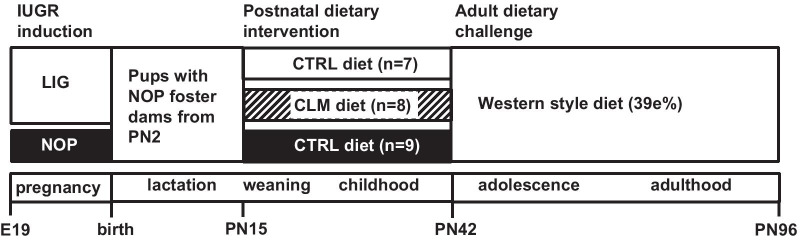
Study design. The group size was dependent on the available blood samples which was affected by the small amounts of the blood samples. The reported group size only included the animals of which all data was available. IUGR: intra-uterine growth restriction, LIG: ligated group, NOP: non-operated group, PN: postnatal day, E: embryonic day

Samples and data used for this study where collected as part of a larger study, of which the phenotypic and physiological data were published previously, including a detailed description of the complete animal study design [7]. The design of the three groups, which were the focus of the present study was as follows, IUGR was induced by bilateral ligation (LIG) of uterine arteries and veins in pregnant Wistar HAN rats (Charles River Wiga Deutschland GmbH). Offspring of non-operated (NOP) dams was included as control group. All pups were nursed by NOP dams, which were fed American Institute of Nutrition-93Growth (AIN-93G) diet until PN15 and then either the control (CTRL) or CLM diet until litters were weaned at PN21 (Fig. 1). Weaned males continued their diet until PN42, followed by a Western-style diet (WSD) challenge (39 en% fat) until PN96. The resulting experimental groups which were the focus of this study were, NOP-CTRL (n = 9), LIG-CTRL (n = 7) and LIG-CLM (n = 8). The early diets (PN15-42) contained 28.3% (w/w) CTRL or CLM infant formula powder and were provided as dough balls in the home cage as described before [7, 8]; for dietary composition see Additional file 1: Table S1. The CLM diet contained large lipid droplets coated with milk-phospholipids [8].

### Physiological parameters

Whole blood was collected after an overnight fast at PN42 and PN96. Blood glucose levels were assessed using ABL 800 FLEX (Radiometer GmbH, Willich, Germany), serum triglyceride levels determined by routine clinical laboratory procedure, and visceral adiposity at PN92 by means of micro-computerized tomography (μCT, LaTheta LCT-100; Aloka Co. LTD., Tokyo, Japan), as described [7].

### Metabolomics analyses

PN42 and PN96 serum metabolite profiles were determined with tandem mass spectrometry using a Metabolomics AbsoluteIDQ® p180 96 AB Sciex Edition Kit (Biocrates, Innsbruck, Austria) as described [11]. Results below limit of detection (LOD) were set at LOD/2, results below lower limit of quantification (LLOQ) were set at (LOD + LLOQ)/2, results above upper limit of quantification (ULOQ) were set to ULOQ. Raw data are reported in Additional file 2.

### Data analyses

Metabolites with > 50% missing values or zero within-group variance for all groups were excluded. On the resulting metabolomics data set, weighted correlation network analysis (WGCNA) was performed for data reduction, leading to co-abundance modules of highly correlated metabolites (EdgeLeap B.V., Utrecht, The Netherlands, Additional file 1 for more details). Data of all experimental groups was used for the WGCNA analyses, including the groups which were not the focus of our research question (see Additional file 3: Fig. S1A for details of all experimental groups). For each module an ‘Eigenprofile’ was calculated, representing overall abundance of the metabolites of the module. Biweight midcorrelations were calculated between ‘Eigenprofiles’ of the modules and physiological parameters at PN96 (PN92 for visceral adiposity). All analyses were performed in R 3.2.2 (2015-08-14) using the WGCNA package 1.46.

### Statistics

Statistical differences between the focus groups were analyzed with GraphPad Prism 7.03 (GraphPad Software. Inc, San Diego, US), using unpaired t-tests for each comparison (NOP-CTRL vs. LIG-CTRL and LIG-CTRL vs. LIG-CLM). Gaussian distribution was tested with the Kolmogorov–Smirnov test. Pearson’s correlations were calculated between individual metabolites and physiological outcomes. Sample size calculations were designed to detect differences in plasma glucose, triglyceride levels as well as differences in body composition in the larger study [7] and was set to at least n = 8 per group. The group size was also dependent on the available blood samples which was affected by the small amounts of the blood samples. The metabolomics analyses are exploratory analyses in this study and as such not included in the power calculations.

## Results

Since the primary aim was to explore potential mechanisms for and identify biomarkers connected to the nutritionally programmed improvements in IUGR on adult visceral adiposity, blood glucose and triglyceride levels [7], we focused on the PN42 metabolomics data, their correlation with adult visceral adiposity, blood glucose and triglyceride levels and compared results between the LIG-CTRL and LIG-CLM (CLM effect), and between NOP-CTRL and LIG-CTRL (LIG effect) groups.

### Data analyses

**Fig. 2:**
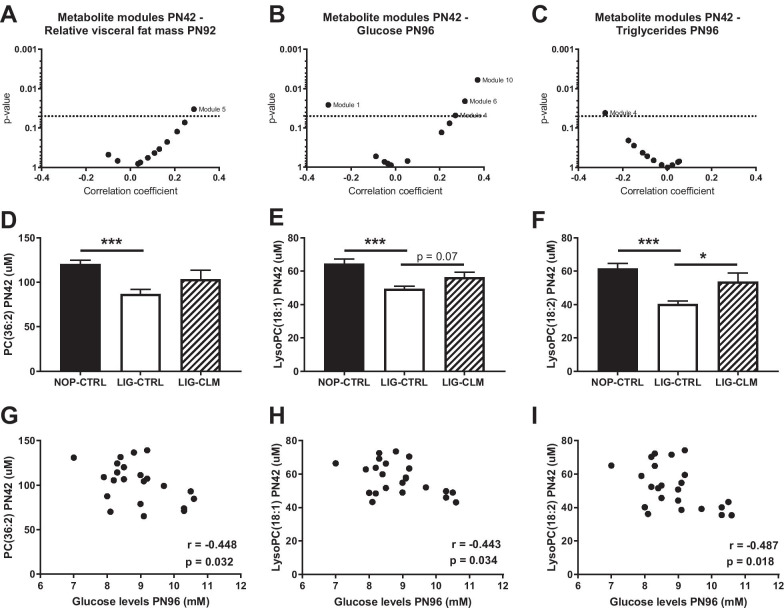
Serum metabolites at PN42 in relation to metabolic parameters at PN96. Correlation between metabolite co-abundance modules at PN42 and **a** relative fat mass at PN92; **b** plasma glucose levels at PN96 and **c** plasma triglyceride levels at PN96; serum **d** PC(36:2), **e** LysoPC(18:1) and **f** LysoPC(18:2) levels at PN42; correlations between glucose levels at PN96 and serum **g** PC(36:2), **h** LysoPC(18:1) and **i** LysoPC(18:2) levels at PN42, with the correlation coefficient r and *p*-value depicted in the graphs. **a**–**c** biweight midcorrelations were based on the calculated ‘Eigenprofiles’ of a metabolite module and metabolic parameters, *p*-values were plotted against the correlation coefficient. Metabolite modules were determined by co-abundance analyses based on data from all groups (n = 57). PC: phosphatidylcholine, PN: postnatal day. * *p* < 0.05; ** *p* < 0.01, *** *p* < 0.001

Out of 192 serum metabolites detected at PN42, 170 met the indicated quality criteria and WGCNA resulted in 11 metabolite modules (Additional file 1: Table S2, and Additional file 4). Correlation analysis between the ‘Eigenprofiles’ of the metabolite modules and later-life relative visceral fat mass, blood glucose and triglyceride levels showed significant correlations between module 5 and relative visceral fat mass (r = 0.287, *p* = 0.033), modules 1, 4, 6 and 10 and blood glucose levels (module 1: r = − 0.303, *p* = 0.026; module 4: r = 0.271, *p* = 0.048; module 6: r = 0.314, *p* = 0.021 and module 10: r = 0.371, *p* = 0.006), and between module 4 and blood triglyceride levels (r = − 0.280, *p* = 0.041; Fig. 2a–c, Additional file 1: Table S3). All those metabolite modules (1, 4, 5, 6 and 10) were subsequently further analyzed.

### Follow up data analyses

Serum levels of the individual metabolites from the indicated modules were tested for significant differences between the focus groups. Three metabolites of module 1, being phosphatidylcholine (PC(36:2)), LysoPC(18:1) and LysoPC(18:2), were decreased in the ligated compared to the non-operated group (LIG-CTRL v. NOP-CTRL: *p* < 0.01, Fig. 2d–f). In turn, levels of LysoPC(18:2) were higher in the LIG-CLM compared to the LIG-CTRL (*p* < 0.05), while LysoPC(18:1) levels tended to be higher (LIG-CTRL v. LIG-CLM: *p* = 0.07). Levels of these three metabolites at PN42 were individually correlated to the blood glucose levels at PN96 (r = − 0.4 and *p* < 0.05 for all metabolites, Fig. 2g–i), but not to blood glucose levels at PN42 (Additional file 3: Fig. S1B-D). Levels of these three metabolites in adulthood (PN96) were not correlated to blood glucose levels at PN96 (Additional file 3: Fig. S1E-G).

## Discussion

In the present study we showed that a diet containing large, milk phospholipid coated lipid droplets restored early-life LysoPC(18:2) levels in a rat model for IUGR. While early-life (PN42) LysoPC(18:2) levels showed no association with early-life glucose levels, we did find that early-life LysoPC(18:2) levels negatively correlated to adult (PN96) basal glucose levels.

In humans, reduced LysoPC(18:2) levels were found in insulin-resistant individuals and appeared years ahead of diagnosis for Type 2 diabetes mellitus (T2D) [12, 13]. How the reduced LysoPC(18:2) levels link with T2D is unknown, but this may include disturbed hepatic phospholipid biosynthesis in the onset of insulin resistance [14]. In the current study, low LysoPC(18:2) levels correlated to elevated adult fasting blood glucose levels in IUGR rats, which might indicate a (pre)diabetic phenotype in early adulthood of fetal growth restricted animals. A direct link between LysoPCs and blood glucose levels is supported by in vitro data showing increased glucose uptake by adipocytes exposed to specific, saturated LysoPCs [15]. However, the absence of a direct correlation between LysoPC(18:2) levels and glucose levels at the same time point, both at PN42 and PN96, in our data does not support such a direct effect for LysoPC(18:2). Instead, the correlation between early-life LysoPC(18:2) levels and adult glucose levels as found in the present study, suggests that set points for adult glucose regulation may be established in early life, thus determining later-life susceptibility to impaired glucose tolerance. Further in vitro and in vivo experiments would be needed to confirm the role of LysoPC(18:2) in long-term regulation of blood glucose levels.

Interestingly, one recent clinical study showed that plasma LysoPC levels are affected by the early-life diet, as blood metabolome of infants fed either human milk, standard IMF or an IMF low in energy and protein, but supplemented with MFGM-fragments showed distinctly different LysoPC levels [16]. In the present study we used a postnatal diet with large lipid globules, coated with milk phospholipids, i.e. MFGM-fragments [8]. Therefore, one might argue that the increased LysoPC(18:2) levels were simply a direct consequence of the higher phospholipid intake. Approximately 30% of phospholipids in the MFGM source used were PCs with a relatively high abundance of C18:2 (~ 10% of total fatty acids [17, 18]). Although a direct link between dietary PC levels and serum LysoPC(18:2) levels cannot be ruled out, the unchanged LysoPC(18:2) levels in the control groups fed CLM in early life (Additional file 1: Table S4), argues against this.

The study had some limitations, e.g. the study was conducted in male offspring only while effects of early life nutrition on the metabolism are sex specific [19] and may therefore be different in female. Furthermore, the results reported are exploratory findings of a larger study, which was not powered for this readout specifically, and thus may be underpowered. Follow up research, preferably in a clinical setting, would be needed to confirm the data, and affirmation of the present results would render LysoPC(18:2) an interesting biomarker, which could also be useful for the clinical setting, i.e. for risk assessment in children born small for gestational age.

Altogether, this study indicates that LysoPC(18:2) levels in early life may be a predictive biomarker for future glucose regulation and T2D risk in the IUGR population, and that these levels can be influenced by an early diet with large, phospholipid coated lipid droplets.

## Supplementary Information

**Additional file 1. Table S1:** Dietary composition. **Table S2:** Metabolites modules with eigenprofiles and number of metabolites per module. **Table S3:** Correlations between relative fat mass, blood glucose and triglyceride levels at PN92-96 and metabolite modules at PN42. **Table S4:** Serum lysoPC(18:2) levels at PN42 in all groups.

**Additional file 2**. Metabolite levels per sample at PN42 and PN98 in all groups.

**Additional file 3**. Study design and correlation between serum metabolite and glucose levels. **A**) study design including all experimental groups used for the WGCNA. Intrauterine growth restriction (IUGR) was induced by bilateral ligation (LIG) in part of the dams, other dams were sham operated (SOP) or were not operated (NOP). 2 days after birth offspring was culled and assigned to NOP foster dams. From postnatal day (PN)15 onwards dams and litters were exposed to the different experimental diets. Offspring was weaned at PN21 and continued feeding the experimental diets until PN42 after which they were challenged with a western style diet. Correlations between plasma glucose and serum **B**) PC(36:2), **C**) LysoPC(18:1) and **D**) LysoPC(18:2) levels at PN42 and between plasma glucose and serum **E**) PC(36:2), **F**) LysoPC(18:1) and **G**) LysoPC(18:2) levels at PN98. E: embryonic day, PC: phosphatidylcholine, PN: postnatal day.

**Additional file 4**. Metabolite module membership of PN42 metabolomics data. Order of metabolites within a module by module membership score.

## Data Availability

The datasets used and/or analyzed during the current study are published as additional files or available from the corresponding author upon reasonable request.

## Abbreviations

AIN-93G: American Institute of Nutrition-93Growth diet
CLM: Complex lipid matrix
TRL: Control
IMF: Infant milk formula
IUGR: Intrauterine growth restricted
LIG: Intrauterine ligation
LLOQ: Lower limit of quantification
LOD: Limit of detection
LysoPC: Lysophosphatidylcholine
MFGM: Milk fat globule membrane
NOP: Non-operated
PC: Phosphatidylcholine
PN: Postnatal day
T2D: Type 2 diabetes mellitus
ULOQ: Upper limit of quantification
WGCNA: Weighted correlation network analysis
WSD: Western-style diet
