# Supplementary material for: The effect of dietary lipid quality in early life on serum LysoPC(18:2) levels and their association with adult blood glucose levels in intrauterine growth restricted rats
Source: Nutr Metab (Lond). 2021 Nov 27;18:101. doi: 10.1186/s12986-021-00614-8 (PMC8627018; doi:10.1186/s12986-021-00614-8)
Supplement: Supplementary file 1 — Additional file 1. Table S1: Dietary composition. Table S2: Metabolites modules with eigenprofiles and number of metabolites per module. Table S3: Correlations between relative fat mass, blood glucose and triglyceride levels at PN92-96 and metabolite modules at PN42. Table S4: Serum lysoPC(18:2) levels at PN42 in all groups. [file 12986_2021_614_MOESM1_ESM.docx]

The effect of dietary lipid quality in early life on serum LysoPC(18:2) levels and the association with adult blood glucose levels in intrauterine growth restricted rats

Andrea Kodde^1^, Mona Mischke^1^, Maryam Rakhshandehroo^1^, Jenny Voggel^2^ Gregor Fink^2^, Eva Nüsken^2^, Manfred Rauh^3^, Eline M. van der Beek^1,4^, Jörg Dötsch^2^, Kai-Dietrich Nüsken^2^

^1^ Danone Nutricia Research, Utrecht, The Netherlands

^2^ Department of Pediatrics and Adolescent Medicine, University of Cologne, Medical Faculty and University Hospital Cologne, Cologne, Germany

^3^ Department of Pediatrics and Adolescent Medicine, University Hospital Erlangen, Erlangen, Germany

^4^ Department of Pediatrics, University Medical Centre Groningen, University of Groningen, Groningen, The Netherlands

**Supplementary method WGCNA analyses**: Weighted co-expression analyses (WGCNA) was applied to identify clusters of highly correlated metabolites. As the sample size of the study was not sufficient to do this analysis per group, all samples of the study were included in the analyses. Signed weighted correlation using biweight midcorrelation was used to construct the network (parameter maxPOutliers = 0.1), a robust version of Pearson correlation. A soft threshold power parameter was fitted based on the criterion of approximately scale-free topology (power parameter 4). Additional file 5 shows a heatmap of the topological overlap matrix. The “blockwiseModules” function from the WCGNA package of R was used to obtain co-abundance modules. With the “blockwiseModules” function topological overlap matrix are constructed, this matrix is clustered using average agglomeration and resulting dendrogram is cut into separate modules using the R package “dynamicTreeCut” (version 1.62). Subsequently, modules are filtered for consistency and size and merged when similar. The “cutHeight” parameter was set to 0.98 for tree cutting and default settings for other parameters. Minimal module size of 5 metabolites was enforced. A correlation

**Additional file 1 Table S1:** Dietary composition

| Diet | | | CTRL | CLM | WSD | AIN93M |
| --- | --- | --- | --- | --- | --- | --- |
| Ingredients | | |  |  |  |  |
| Casein | | g/kg | 168 | 168 | 200 | 140 |
| CTRL-IMF | | g/kg | 283 | - | - | - |
| CLM-IMF | | g/kg | - | 283 | - | - |
| Cornstarch, pre-gelatinized | | g/kg | 300 | 300 | 100 | 466 |
| Maltodextrin | | g/kg | 101 | 101 | 60 | 155 |
| Sucrose | | g/kg | 70 | 70 | 330 | 100 |
| Cellulose powder B800 | | g/kg | 39 | 39 | 50 | 50 |
| L-Cystine | | g/kg | 2.2 | 2.2 | 3 | 1.8 |
| Vitamin premix | | g/kg | 7 | 7 | 10 | 10 |
| Mineral & trace element premix | | g/kg | 28 | 28 | 35 | 35 |
| Choline chloride | | g/kg | 1.9 | 1.9 | 2.5 | 2.2 |
| Pork lard | | g/kg | - | - | 170 | - |
| Soybean oil | | g/kg | - | - | 30 | 40 |
| Nutritional composition | | |  |  |  |  |
| Carbohydrates (total) | | g/kg | 500 | 500 | 437 | 567 |
|  | Starch | g/kg | 268 | 268 | 98 | 455 |
|  | Sugar | g/kg | 232 | 232 | 339 | 112 |
| Protein (total) | | g/kg | 177 | 177 | 177 | 123 |
| Fat (total) | | g/kg | 70 | 70 | 211 | 41 |
|  | SFA | g/kg | 28.7 | 29.7 | 41 | 5.9 |
|  | MUFA | g/kg | 26.5 | 26.1 | 42.3 | 10.3 |
|  | PUFA | g/kg | 11.5 | 11 | 13.2 | 23.5 |
|  | LA:ALA |  | 5.4 | 5.3 | 9.5 | 7.5 |
|  | Phospholipids | g/kg | 0.09 | 1.13 | - | - |
|  | Cholesterol | mg/kg | 4.8 | 15.7 | - | - |
| Fibre | | g/kg | 49 | 49 | 50 | 50 |
| Ash | | g/kg | 33 | 33 | 31 | 30 |

**Additional file 1 Table S2:** Metabolites modules with eigenprofiles and number of metabolites per module.

| Module: | Eigenprofile | Number of metabolites |
| --- | --- | --- |
| Module 1 | 0,665 | 26 |
| Module 2 | 0,603 | 18 |
| Module 3 | 0,705 | 17 |
| Module 4 | 0,767 | 13 |
| Module 5 | 0,716 | 11 |
| Module 6 | 0,584 | 11 |
| Module 7 | 0,545 | 9 |
| Module 8 | 0,641 | 8 |
| Module 9 | 0,658 | 7 |
| Module 10 | 0,520 | 6 |
| Module 11 | 0,742 | 5 |

**Additional file 1 Table S3:** Correlations between relative fat mass, blood glucose and triglyceride levels at PN92-96 and metabolite modules at PN42.

| Metabolite module PN42 | Relative fat mass PN92 | | Glucose PN96 | | Triglyceride PN96 | |
| --- | --- | --- | --- | --- | --- | --- |
|  | Correlation coefficient | p-value | Correlation coefficient | p-value | Correlation coefficient | p-value |
| Module 1 | 0,035 | 0,800 | **-0,303** | **0,026** | -0,025 | 0,859 |
| Module 2 | 0,211 | 0,123 | 0,055 | 0,692 | -0,061 | 0,659 |
| Module 3 | 0,166 | 0,225 | -0,049 | 0,727 | 0,048 | 0,729 |
| Module 4 | 0,131 | 0,340 | **0,271** | **0,048** | **-0,280** | **0,041** |
| Module 5 | **0,287** | **0,033** | -0,088 | 0,527 | 0,023 | 0,869 |
| Module 6 | 0,244 | 0,071 | **0,314** | **0,021** | -0,111 | 0,424 |
| Module 7 | -0,099 | 0,472 | -0,034 | 0,808 | 0,054 | 0,697 |
| Module 8 | 0,109 | 0,428 | 0,244 | 0,076 | -0,175 | 0,206 |
| Module 9 | -0,057 | 0,678 | 0,208 | 0,130 | 0,001 | 0,993 |
| Module 10 | 0,045 | 0,743 | **0,371** | **0,006** | -0,090 | 0,518 |
| Module 11 | 0,079 | 0,567 | -0,020 | 0,883 | -0,150 | 0,278 |

Significant values in bold. Correlation coefficients are biweighted midcorrelations.

**Additional file 1 Table S4:** Serum lysoPC(18:2) levels at PN42 in all groups.

| µM | NOP-CTRL | SOP-CTRL | LIG-CTRL | NOP-CLM | SOP-CLM | LIG-CLM |
| --- | --- | --- | --- | --- | --- | --- |
| LysoPC(18:2) | 61.6 ± 9.3 | 54.6 ± 12.7 | 40.5 ± 4.1 | 54.8 ± 16.3 | 46.0 ± 9.3 | 53.8 ± 14.4 |
