# Supplementary material for: The effect of dietary lipid quality in early life on serum LysoPC(18:2) levels and their association with adult blood glucose levels in intrauterine growth restricted rats
Source: Nutr Metab (Lond). 2021 Nov 27;18:101. doi: 10.1186/s12986-021-00614-8 (PMC8627018; doi:10.1186/s12986-021-00614-8)

**Additional file 3**: Study design and correlation between serum metabolite and glucose levels. **A)** study design including all experimental groups used for the WGCNA. Intrauterine growth restriction (IUGR) was induced by bilateral ligation (LIG) in part of the dams, other dams were sham operated (SOP) or were not operated (NOP). 2 days after birth offspring was culled and assigned to NOP foster dams. From postnatal day (PN)15 onwards dams and litters were exposed to the different experimental diets. Offspring was weaned at PN21 and continued feeding the experimental diets until PN42 after which they were challenged with a western style diet. Correlations between plasma glucose and serum **B)** PC(36:2), **C)** LysoPC(18:1) and **D)** LysoPC(18:2) levels at PN42 and between plasma glucose and serum **E)** PC(36:2), **F)** LysoPC(18:1) and **G)** LysoPC(18:2) levels at PN98. E: embryonic day, PC: phosphatidylcholine, PN: postnatal day.


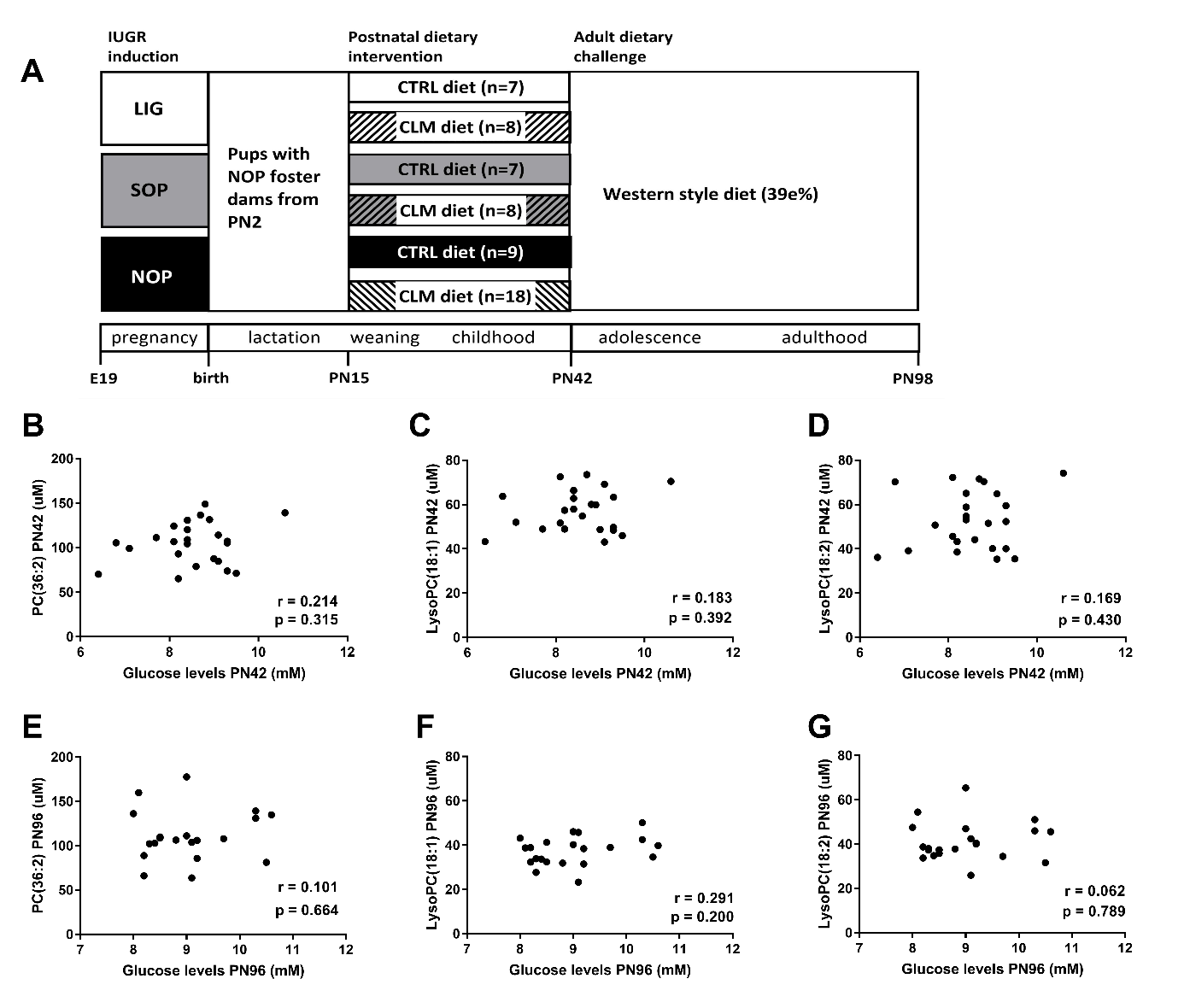

Supplement: Supplementary file 3 — Additional file 3. Study design and correlation between serum metabolite and glucose levels. A) study design including all experimental groups used for the WGCNA. Intrauterine growth restriction (IUGR) was induced by bilateral ligation (LIG) in part of the dams, other dams were sham operated (SOP) or were not operated (NOP). 2 days after birth offspring was culled and assigned to NOP foster dams. From postnatal day (PN)15 onwards dams and litters were exposed to the different experimental diets. Offspring was weaned at PN21 and continued feeding the experimental diets until PN42 after which they were challenged with a western style diet. Correlations between plasma glucose and serum B) PC(36:2), C) LysoPC(18:1) and D) LysoPC(18:2) levels at PN42 and between plasma glucose and serum E) PC(36:2), F) LysoPC(18:1) and G) LysoPC(18:2) levels at PN98. E: embryonic day, PC: phosphatidylcholine, PN: postnatal day. [file 12986_2021_614_MOESM3_ESM.docx]
